# Intein-mediated thyroid hormone biosensors: towards controlled delivery of hormone therapy

**DOI:** 10.3389/fsysb.2024.1270071

**Published:** 2024-04-03

**Authors:** Quim Martí-Baena, Andreu Pascuet-Fontanet, Tomas Berjaga-Buisan, Miriam Caravaca-Rodríguez, Jaume Puig-Costa-Jussà, Avencia Sanchez-Mejias, Dimitrije Ivančić, Sira Mogas-Díez, Marc Güell, Javier Macia

**Affiliations:** ^1^ Department of Medicine and Life Sciences, Universitat Pompeu Fabra, Barcelona, Spain; ^2^ Department of Information and Communications Technologies, Universitat Pompeu Fabra, Barcelona, Spain

**Keywords:** synthetic biology, mathematical modeling, hypothyroidism, thyroid hormones, biosensor, intein

## Abstract

Although blood sampling and medical imaging are well-established techniques in clinical diagnostics, they often require long post-processing procedures. Fast and simple quantification of signaling molecules can enable efficient health monitoring and improve diagnoses. Thyroid hormones (THs) treatment relies on trial-and-error dose adjustments, and requires constant tracking via blood tests. Thus, a fast and reliable method that can constantly track THs levels could substantially improve patient quality of life by reducing their visits to doctors. Synthetic biosensors have shown to be inexpensive and easy tools for sensing molecules, with their use in healthcare increasing over time. This study describes the construction of an engineered THs bacterial biosensor, consisting of a split-intein-based TH receptor ligand binding domain (LBD) biosensor that reconstitutes green fluorescence protein (GFP) after binding to TH. This biosensor could quantitatively measure THs concentrations by evaluating fluorescence intensity. *In vitro* sensing using *Escherichia coli* produced GFP over a wide dynamic range. The biosensor was further optimized by adding a double LBD, which enhanced its dynamic range until it reached healthy physiological conditions. Moreover, a mathematical model was developed to assess the dynamic properties of the biosensor and to provide a basis for the characterization of other intein-mediated biosensors. This type of biosensor can be used as the basis for novel treatments of thyroid diseases and can be adapted to measure the concentrations of other hormones, giving rise to a series of mathematically characterized modular biosensors.

## 1 Introduction

DNA electrochemical biosensors enable fast and simple quantification of human-derived metabolites and hold great potential as new diagnostic tools (Kowalczyk, 2020). Moreover, when combined with cell engineering, the use of human cell-derived receptors is promising for the development of new clinical applications, such as the chimeric antigen receptors used in CAR-T therapy (Sadelain, 2016). At the same time, skin-interfaced biosensors integrated into wearable and implantable systems is under development and promises to revolutionize the diagnostics field (Dervisevic et al., 2020). With that, telemedicine may take advantage of these synthetic receptors to monitor patient health status (Ekeland et al., 2010).

**FIGURE 1:**
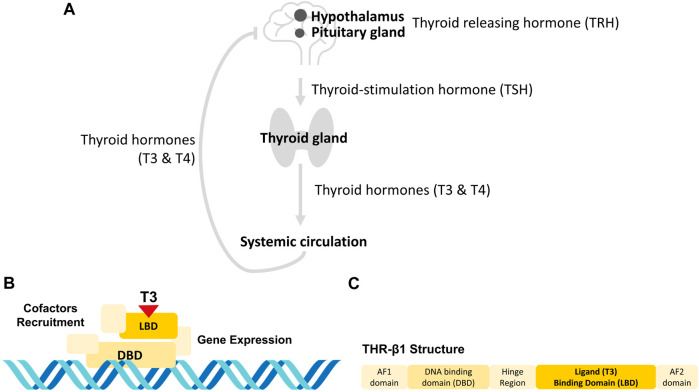
THs sensing at the body **(A)** and molecular **(B, C)** levels. **(A)** *The hypothalamic-pituitary-thyroid axis*. When the hypothalamus senses low levels of THs, it releases TRH, which activates the pituitary gland to release TSH. TSH, in turn, stimulates the thyroid gland to produce THs that are released to the systemic circulation. When the hypothalamus senses that levels of TH are sufficient, it stops releasing TRH, so that no more TH are produced (Dietrich et al., 2012). **(B)** *THR activation molecular mechanism*. THRs are a group of nuclear proteins that can bind to specific hormones through their LBDs, which interact with T3 (Yen, 2001). This is followed by the binding of the THR to a specific sequence using its DBD, and the recruiting of proteins to unpack the chromatin and induce gene transcription (Flamant et al., 2017). **(C)** *Human THR-* *β*_1_ *isoform.* The human THR-*β*_1_ contains a hinge region between the LBD and DBD that provides the receptor with enough flexibility to change between its active and inactive forms. The LBD, DBD and hinge region are embedded by the activation functions (AF) AF1 and AF2 at the N- and C-termini, respectively. These AFs allow the THR-*β*_1_ receptor to bind to coactivators to initiate transcriptional activation of target genes.

Thyroid disease is characterized by a dysregulation of the hypothalamic-pituitary-thyroid axis (e.g., Figure 1A). When the hypothalamus senses low levels of thyroid hormones (THs), it secretes thyroid releasing hormone (TRH), which stimulates the pituitary gland to secrete thyroid-stimulating hormone (TSH) (Dietrich et al., 2012). TSH, in turn, stimulates the thyroid gland to produce THs, which are released into the systemic circulation. When the hypothalamus senses that TH levels are sufficient, it stops releasing TRH, thus turning off the production of THs (Dietrich et al., 2012). One of the most common thyroid diseases is hypothyroidism, which affects 5% of the worldwide population and is characterized by the lack of production of THs (Chiovato et al., 2019). Hypothyroidism is usually treated by the oral administration of synthetic thyroxine (T4), which is metabolized to its active triiodothyronine form (T3) in peripheral tissues (Dietrich et al., 2012; Fliers et al., 2018). However, some patients present malabsorption of oral synthetic T4 and/or require to complement the treatment with T3 administration, for which subcutaneous administration seems a successful treatment option (Groener et al., 2013; Idrees et al., 2019). In any case, THs doses are initially adjusted by trial-and-error, with several analyses and visits to the doctor, and require indefinite follow-ups to readjust these doses over time (American Thyroid Association et al., 2004).

A portable sensing system to quantify THs would be extremely useful for providing patient-specific doses and reducing the number of visits to the doctor. This may be provided by a system that mimics the detection of THs through thyroid hormone receptors (THRs), a group of nuclear proteins that can bind to specific hormones through their ligand binding domains (LBDs), which interact with T3 (Yen, 2001). This results in the binding of THRs to a specific sequence of the THs using their DNA binding domains (DBD), recruiting proteins to unpack chromatin and inducing gene transcription (see Figures 1B,C) (Flamant et al., 2017).

Combinations of regulatory components may mimic existing biological receptors (Khalil and Collins, 2010). THR-*α*_1_ and THR-*β*_1_ have already been successfully expressed in *Escherichia coli* (*E. coli*). More specifically, inteins, which have been used in synthetic biology and therapeutics for the last 10 years, may sense T3 in bacterial cells (Gierach et al., 2012; Wang et al., 2022). Inteins are a family of intervening proteins responsible for post-translational protein splicing. Once their mechanism is activated, they can remove themselves from the peptide chain by joining the two remaining portions on both sides (Wood et al., 1999). The great advantage of this process is that it is spontaneous, with neither external factors nor energy sources, such as ATP, needed to splice the protein (Shah and Muir, 2014).

These biosensors were not designed for clinical use, as they were based on the growth effect of engineered cells (Gierach et al., 2012). These biosensors utilize the linkage of thymidylate synthase activity, which is basic for *E. coli* survival, with the *Mycobacterium tuberculosis* (*Mtu*) RecA intein incorporating the THR LBD into the splicing domain. Cell growth was therefore associated with the amount of T3 present in the cell growth medium. Broader applications therefore require another perspective (Brooks and Alper, 2021), in which whole cell or enzymatic biosensors can be stabilized inside cellulose based composites (Mohammadi et al., 2023).

To address this need, it was hypothesized that linking intein activity with a fluorescent protein would result in a TH biosensor that could be connected with electronic systems for clinical and experimental applications (Beyenal et al., 2004). In addition, fluorescence provides a direct link to protein-splicing activity. Thus, fluorescence-based biosensors would be better suited for sensing a wide range of TH concentrations than optical density (OD)-based biosensors.

Several studies have suggested that biosensor design can be based on the splitting of green fluorescent proteins (GFPs) by different inteins, but fewer studies have assessed the usage of the *Mtu* RecA intein to split GFP (Ozawa et al., 2000). On top of that, those few studies were based on splitting the enhanced version of GFP (eGFP) (Tornabene et al., 2019), although improved versions such as the superfolder GFP (sfGFP) could allow for faster and more robust folding of the protein (Pédelacq et al., 2006). However, the splicing of GFPs with the *Mtu* RecA intein should first be validated with eGFP.

In addition, a minimal and more stable version of the *Mtu* RecA intein is now available. This version is achieved by introducing the V67L mutation, which may be useful in reducing the complexity of the construct and enhancing its stability (Hiraga et al., 2005). The present study describes the design of a quantitative intein-mediated T3 (IMT3) biosensor that emits GFP fluorescence proportionally to the ligand concentration in the media.

## 2 Materials and methods

Three different versions of the IMT3 biosensor were developed. The first was an enhanced IMT3 biosensor (eIMT3), a basic version with eGFP; the second was a superfolder IMT3 biosensor (sfIMT3), an improved version with sfGFP; and the third was a double IMT3 biosensor (dIMT3), a version with two tandem LBDs, resulting in enhanced specificity and sensitivity of the biosensor. A generic mathematical model was created to assess the activity of the sfIMT3 biosensor, and experimental data were obtained to fit the parameters of the model.

### 2.1 Intein-mediated T3 biosensors design

The IMT3 biosensors were based on a chimeric protein structure, consisting of THR-*β*_1_ LBD fused between the first 110 residues, and the final 58 residues of the full-length minimal, more stable version of the *Mtu* RecA intein, with a spliced reporter at each end of the construct (i.e., half of the reporter on each side (Wood et al., 1999; Hiraga et al., 2005)). This construct showed post-transcriptional splicing activity following selective binding of the LBD to T3 and T4, making the spliced reporter functional.

The spliced reporter was used to create two variants of the IMT3 biosensor: eIMT3 and sfIMT3. eIMT3 used eGFP (FPbase ID: R9NL8) split between residues 70 and 71 as a reporter, whereas sfIMT3 used sfGFP (FPbase ID: B4SOW), split between residues 69 and 70, identical to the amino acid split point of eGFP (Tornabene et al., 2019). eIMT3 was designed for the single purpose of proving that *Mtu* RecA was able to splice GFP, while sfIMT3 was designed for biosensor’s characterization.

To avoid the formation of inclusion bodies by aggregation of the protein chimera, the solubility tag Fh8 was fused to the N-terminal of its sequence, and a FLAG tag was fused to its C-terminal (Gangopadhyay et al., 2003; Costa et al., 2014). The recombinant protein was arranged with the BBa_J23100 synthetic promoter, the BBa_B0034 RBS, and the BBa_B0014 terminator from the Repository of Standard Biological Parts (RSBP)^1^. The eIMT3 and sfIMT3 constructs were synthesized in cloning vectors by Integrated DNA Technologies. Sequences were made available at the RSBP for eIMT3 (Part ID: BBa_K3484003) and sfIMT3 (Part ID: BBa_K3484001) and are also found in the Supplementary Section S1.2. The plasmid constructs are shown in Supplementary Figure S1A, B in the, Section S1.1.

Because the physiological concentrations of T3 in the bloodstream are low, ranging from 7.47 ⋅ 10^–11^–2.87 ⋅ 10^–9^ M, and because increasing the number of receptors enhances the ability of a biosensor to detect lower ligand concentrations, the dIMT3 version with two THR-*β*_1_ LBDs was generated (Dickson, 1990; Gonzalez-Flo et al., 2020). This version was created by codon optimizing a second THR-*β*_1_ LBD receptor and inserting it in tandem between the prior THR-*β*_1_ LBD and the final 58 residues of the *Mtu* RecA intein of sfIMT3. The second LBD was added through Golden Gate assembly by PCR, using six primers designed to include BsaI restriction sites on the biosensor sequence (see Supplementary Section S1.3). The dIMT3 plasmid construct is illustrated in Supplementary Figure S1C, Section S 1.1.

### 2.2 Phenotypic characterization

BL21 cells transformed by electroporation were cultured in the presence of the IMT3 biosensor ligands T3 and T4. To analyze the performance of the biosensors, protein expression was assessed by Western blotting, and the fluorescence of the IMT3 biosensors were measured at various ligand concentrations.

The Rosetta^TM^(DE3)pLysS *E. coli* B strain F- ompT hsdSB (rB- mB-) gal dcm (DE3) pLys-SRARE (CamR) (Novagen) was transformed with eIMT3, sfIMT3 and dIMT3 by electroporation (protocols for the preparation of electrocompetent cells and their transformation by electroporation are available in the Supplementary Section S5.1, 5.2). Transformed cells were grown in Lysogeny Broth (LB) at 37 °C overnight from single colonies to reach the early stationary phase, and selected with appropriate antibiotics (Carbenicillin 25 *μ*g/mL, Sigma, USA)

Selective ligands used to characterize T3 (3,3′,5-triiodo-L-thyronine sodium salt, 95%, Sigma) and T4 (3,3′,5,5”-Tetrayodo-L-tironina, Certified Reference Material, Sigma Aldrich, USA) were dissolved to 100 mM in dimethyl sulfoxide (DMSO) (99,9%, Sigma Aldrich) and stored at −80 C. The concentration of DMSO did not significantly affect study results (Supplementary Section S4).

For Western blotting, saturated eIMT3-transformed cell cultures (8 mL) with and without 2x concentrations of T3 in the media were washed twice with ice-cold S30 buffer, resuspended in 2 mL of ice-cold S30 buffer, and aliquoted into 1 mL microcentrifuge tubes. The cells were placed on ice and lysed by sonication with a Branson Digital Sonifier 250 and a Sonifier Sound Enclosure by applying three cycles of 30 s of 0.2 J/s sonication and 90 s of rest. The lysates were centrifuged at 14,000 rpm for 15 min at 4°C and aliquoted in 4:1 concentrations of loading buffer, with the mixtures incubated at 95 °C for 5 min. The samples were loaded onto SDS-PAGE gels, electrophoresed, and transferred to nitrocellulose membranes. The protocol is fully described in the Supplementary Section S5.3.

sfIMT3 and dIMT3 receptors were characterized in cells treated with different concentrations of T3 and T4. Solutions with increasing concentrations of each ligand were prepared in fresh LB + Carbenicillin 25 *μ*g/mL. Cells transformed with sfIMT3 and dIMT3 were grown overnight until saturation. A 500 *μ*L aliquot of each culture was inoculated into 15 mL of LB and the cells were cultured until they reached an *OD*_600_ of 0.4–0.6. A 100 *μ*L aliquot of cultured cells was added to each well of a 96 well black/clear bottom plate (ThermoFisher, USA), along with 100 *μ*L of the 2x ligand solutions. The 96 well plates were incubated in a Plate Reader (TECAN INFINITE M NANO+) at 37 °C with linear shaking at an amplitude of 1.5 mm for 48 h. Every 20 min, *OD*_660_ and GFP fluorescence (*λ*_*excitation*_: 478 nm, *λ*_*emission*_: 517 nm) were measured. Controls for the fluorescence of the medium consisted of 200 *μ*L/well of fresh LB containing Carbenicillin 25 *μ*g/mL.

### 2.3 Mathematical characterization

#### 2.3.1 Mathematical model

Biosensors that work at the translational level were created to show system interactions and reactions between proteins. Generally, cells produced a receptor protein, *P*, at a rate of *α*_*P*_. *P* is degraded at a rate of *δ*_*P*_, but its free form also is reduced upon binding ligand *A* at a rate of *β*_1_, conforming to the precursor form [*PA*] of the reporter protein *R*. [*PA*] achieves its functional state *R* at rate of *β*_2_. *R* also undergoes basal production and degradation at rates of *α*_*R*_ and *δ*_*R*_, respectively. These interactions are summarized below, and the parameters are also described in Table 1. For further simplification, *β* was set as equal to *β*_1_ *β*_2_.

**TABLE 1:** Parameters involved in system interactions. The first column shows the generic parameters of the model, whereas the second column defines specific parameters involved in IMT3 biosensor sensing and reporting activities.

	Generic descriptor	IMT3 biosensor characterization
*P*	Receptor protein concentration	IMT3 biosensor variant concentration
*A*	Ligand concentration	T3/T4 concentration
[*PA*]	Receptor/ligand complex concentration	Active IMT3 biosensor concentration
*R*	Reporter protein concentration	Functional GFP concentration
*α*_*P*_	Production rate of P	Production rate of the IMT3 biosensor variant
*β*_1_	Binding affinity of P to A	Binding rate of T3/T4 to the IMT3 biosensor
*δ*_*P*_	Degradation rate of P	Degradation rate of the IMT3 biosensor
*α*_*R*_	Production rate of R	Production rate of functional GFP
*β*_2_	Fraction of functional PA	Fraction of correctly spliced GFP
*δ*_*R*_	Degradation rate of R	Degradation rate of functional GFP


$$Ø\overset{\alpha_{P}}{\to }P$$

$$P\overset{\delta_{P}}{\to }Ø$$

$$P+A\overset{\beta_{1}}{\to }[PA]$$

$$[PA]\overset{\beta_{2}}{\to }R$$

$$Ø\overset{\alpha_{R}}{\to }R$$

$$R\overset{\delta_{R}}{\to }Ø$$

Based on system interactions, the concentration evolution of the generated proteins (i.e., *P* and *R*) over time can be determined using ordinary differential equations (ODEs) (see Eqs 1, 2).

$$\frac{dP}{dt}=\alpha_{P}-\beta_{1}PA-\delta_{P}P$$ (1)

$$\frac{dR}{dt}=\alpha_{R}+\beta_{2}\beta_{1}PA-\delta_{R}R$$ (2)

This represents a full description of the species interacting in the system, which depends on the concentration of *A*. Then, the transfer function that links the measured reporter protein *R* to the concentration of ligand *A* can be derived from the ODEs (see Eq. 3 and Supplementary Section S3.3 for the full derivation).

$$R^{*}=\frac{\delta_{P}\alpha_{R}+A\left(\alpha_{P}\beta +\beta_{1}\alpha_{R}\right)}{\beta_{1}\delta_{R}A+\delta_{P}\delta_{R}}$$ (3)

#### 2.3.2 Metrics derivation

**FIGURE 2:**
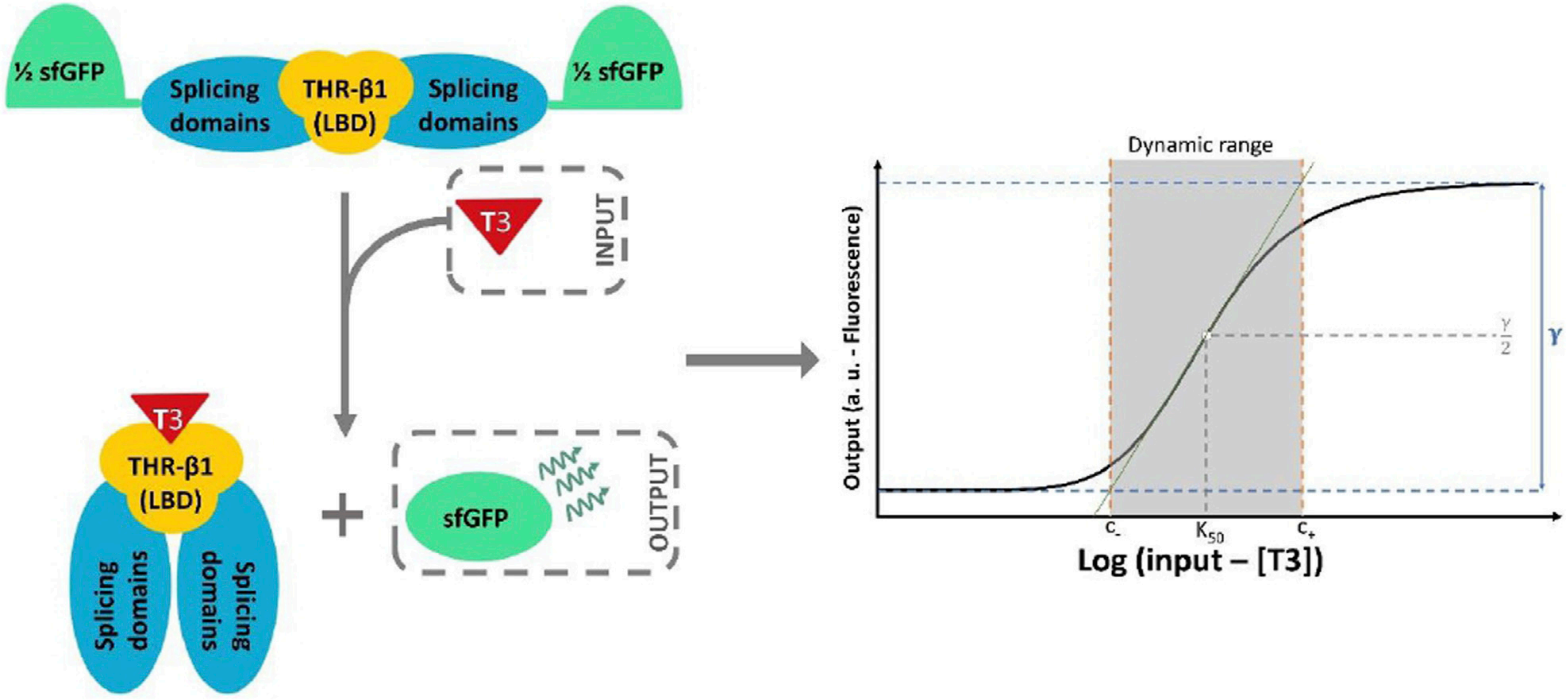
Schematic representation of sfIMT3 activity upon binding T3. sfIMT3 consists of the THR-*β*_1_ LBD fused with the splicing domains of the *Mtu* RecA intein, with spliced sfGFP acting as a reporter (see Section 2.1 for more information). The binding of T3 to the LBD results in the reconstruction of the sfGFP by the splicing domains of the inteins. This system creates a Hill shaped transfer function that connects the T3 concentration in the media to the fluorescence emitted by the sensor cells. In addition, a geometrical definition of the amplitude of a general biosensor (*γ*) and its dynamic range is shown. The boundaries are indicated by the points at which the green tangential line intersects with the maximum and minimum output values of the biosensor (see Eqs 7–9).

sfIMT3 and dIMT3 biosensors were characterized by considering four important metrics derived from the transfer function (Eq 3): amplitude, signal-to-noise ratio (SNR), sensitivity, and dynamic range (Gierach et al., 2012; Gonzalez-Flo et al., 2020). Amplitude can be described as the ratio of the maximum to the minimum output values (see Eq. 4; Figure 2 for a graphic representation of the amplitude). Biosensors characterized by Hill functions with higher amplitudes are desirable because they allow for either a higher sensitivity at a constant dynamic range or a higher dynamic range at a constant sensitivity.

$$\gamma =\frac{\alpha_{P}\beta_{2}}{\delta_{R}}$$ (4)

SNR is a measure of the signal power with respect to the power of the background noise (Carlson and Crilly, 2010). Because biological systems have an intrinsic noise, a biosensor with a higher SNR will decrease the uncertainty of a measurement. SNR can be defined as the ratio of the mean square of a measurement (*μ* ^2^) with respect to its variance (*θ* ^2^) (see Eq 5).

$$SNR=\frac{\mu^{2}}{\theta^{2}}$$ (5)

The sensitivity of a biosensor can be defined as the minimum input parameter that can generate a detectable output (McGrath and Scanaill, 2013). The maximum sensitivity of a sensor with a Hill transfer function is the input value that produces half the maximum output value. For these biosensors, this point can be defined by the expression $K_{0.5}=\frac{\delta_{P}}{\beta }$ . Sensitivity (*ξ*) was calculated by the weighted derivative of the transfer function (Eq 6).

$$\xi =\frac{dR^{*}}{dA}\frac{A}{R}=\frac{A}{R}\frac{\alpha_{P}\delta_{P}\beta }{\delta_{R}{\left(\delta_{P}+\beta_{1}A\right)}^{2}}$$ (6)

Finally, the dynamic range is defined as the range of input values at which the sensor produces a change in the output value (McGrath and Scanaill, 2013). Regions outside this range cannot be sensed because, in this case, the fluorescence has no significant dependence on the TH concentration. The dynamic range can be mathematically characterized by the upper and lower bounds *C*_+_ and *C*_−_. To define the two bounds, the tangent line *f* to the point *K*_0.5_ in the transfer function can be drawn if C is considered to be the logarithm of T3 concentration (see Eq. 7; Supplementary Material S1 for full details of the equation derivation) (Gonzalez-Flo et al., 2020). *C*_+_ and *C*_−_ can be described as the intersection between the tangent line and the minimum and maximum amplitude values of the Hill function (see Eqs 8, 9, respectively). Figure 2 shows the geometrical explanation of this approximate definition of the dynamic range.

$$f=\frac{\alpha_{P}\beta ln\left(10\right)}{4\delta_{R}\beta_{1}}C+\frac{\alpha_{R}}{\delta_{R}}+\frac{\alpha_{P}\beta }{2\delta_{R}B}\left(1-\frac{ln\left(10\right)log\left(\frac{\delta_{P}}{\beta_{1}}\right)}{2}\right)$$ (7)

$$C_{-}=log\left(\frac{\delta_{P}}{\beta_{1}}\right)-\frac{2}{ln\left(10\right)}$$ (8)

$$C_{+}=log\left(\frac{\delta_{P}}{\beta_{1}}\right)+\frac{2}{ln\left(10\right)}$$ (9)

#### 2.3.3 Fitting to experimental data

Experimental data collected with TECAN i-control were analyzed with Microsoft Excel 2019 and Python 3.5.2 with Numpy 1.25, Pandas 2.0.3, SciPy 1.11.1, Statistics 3.4 and Matplotlib 3.7.2 libraries. Experimental data was normalized by subtracting the fluorescence and *OD*_660_ of the wells to the LB + Carbenicillin blank. To account for cell growth, the subtracted fluorescence was divided by the subtracted *OD*_660_ (Eq 10). Finally, for aesthetic purposes, for each transfer function data was subtracted by its minimal value. To avoid an underestimation of the SNR, this final step was not done when calculating the SNR (Eq 5). The transfer function (Eq 3) was fitted to the steady states of the experimental data at 30 h after TH exposure using the least-squares method (see Supplementary Figure S2, for the time dynamics of the IMT3 biosensor responses). The *p*-values of the fluorescence data were assessed using Welch’s *t*-test, as the compared groups differed in variance, with significance defined as *α* = 0.05. The *p*-values of the Western blotting data were assessed by Student’s t-tests, due to the small sample sizes. The effect sizes were calculated using Hedges’ *g* measure. The code used to fit experimental data to the model is available in a GitHub repository^2^.

$$Fluorescence/OD=\frac{GFP-GFP_{\mathrm{Blank}}}{OD-OD_{\mathrm{Blank}}}$$ (10)

## 3 Results

### 3.1 Cells express the IMT3 biosensor and GFP is reconstituted after splicing

**FIGURE 3:**
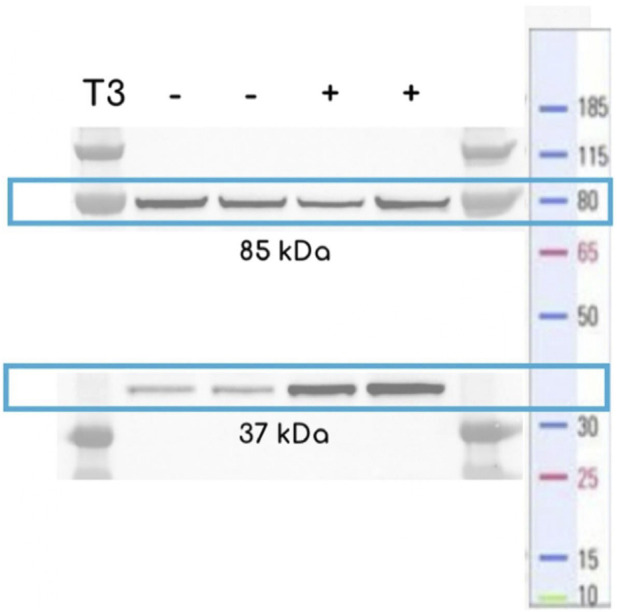
Western blotting of BL21 expressing eIMT3 using a SDS-PAGE gel. Cells were grown in fresh LB media + Kanamycin 25 *μ*g/mL with 10^–4^ M T3 (+) or in the absence of T3 (−) overnight until reaching saturation (Western blot protocol can be found in Section 2.2). Controls show the presence of the entire construct (85 kDa), whereas cells treated with T3 show higher concentrations of the eGFP after splicing (37 kDa).

The biochemical functionality of the biosensors introduced in this study was evaluated by Western blotting analyses of the eIMT3 construct, determining the co-expression of the chimera and its linkage with T3. Cells exhibited constitutive expression of the complete protein, as shown by the protein band at 85 kDa (Figure 3). Splicing activity was confirmed by the presence of a 37 kDa protein band, corresponding to the joined halves of the eGFP plus tags. Although the intensity of the protein bands was significantly higher in cultures treated with and without T3 (*p* = 0.02, *g* = 8.49), splicing was observed in both the presence and absence of T3. Having confirmed the splicing activity of GFPs with *Mtu* RecA in the eIMT3 biosensor, characterization of sfIMT3 and dIMT3 biosensors was performed, as detailed in the following subsections.

### 3.2 sfIMT3 fits the mathematical model, but dIMT3 does not

**FIGURE 4:**
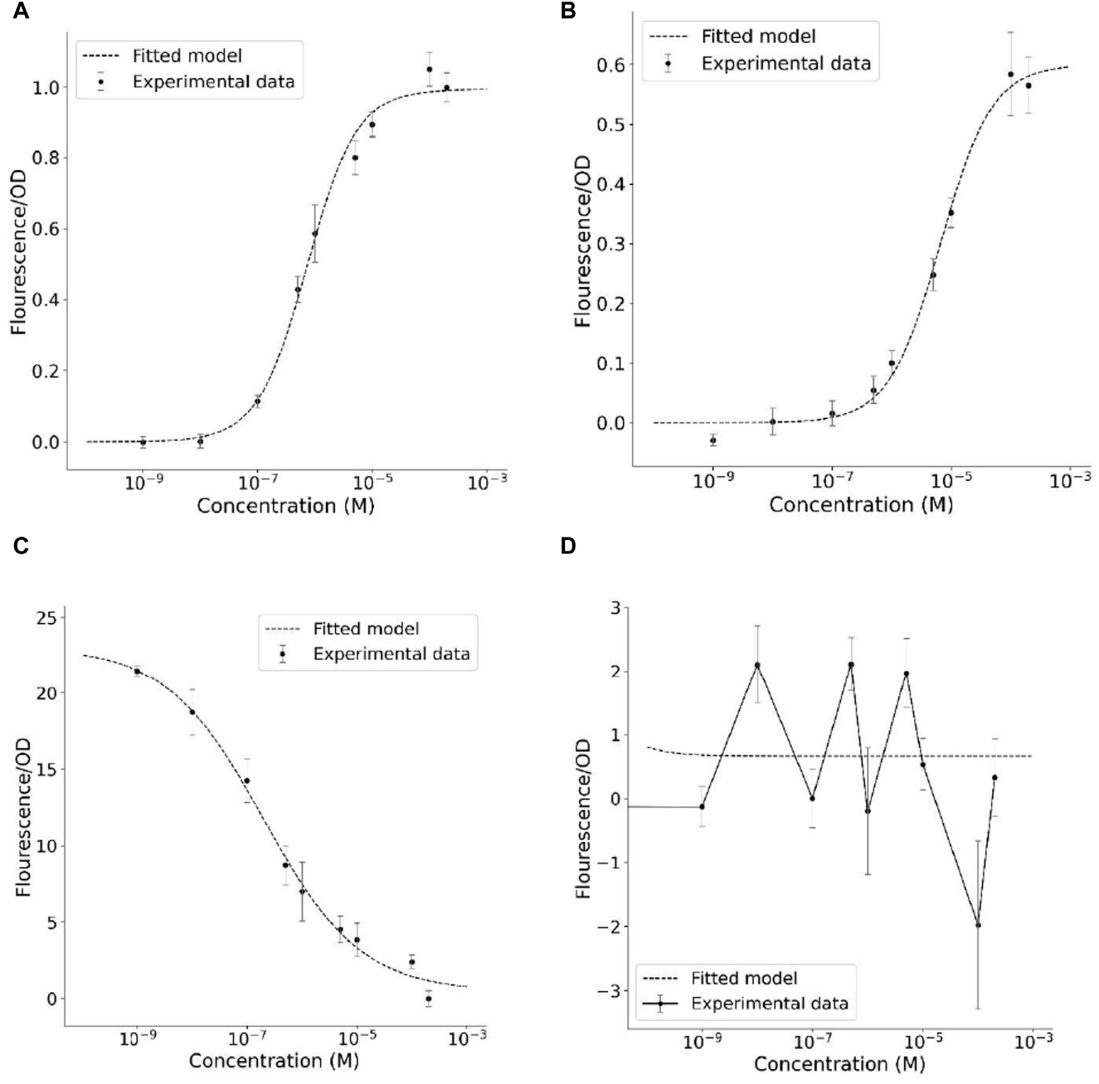
Transfer functions correlating the emitted fluorescence with the amount of thyroid hormone of the biosensors **(A)** sfIMT3 induced with T3 **(B)** sfIMT3 induced with T4 **(C)** dIMT3 induced with T3 **(D)** dIMT3 induced with T4. Cells were grown in fresh LB media + Carbenicillin 25 *μ*g/mL overnight until they attained an *OD*_600_ of 0.4–0.6. A 100 *μ*L aliquot of cultured cells was added to each well of a 96 well black/clear bottom plate, along with 100 *μ*L of the 2x ligand solutions. Experimental data represents the mean value of six replicates per TH concentration at 30 h after TH exposure, with error bars showing its standard error. The transfer functions were fitted using the least-squares method.

The transfer functions of the biosensors were assessed by evaluating the relationship between fluorescence and the concentrations of T3 and T4 agonists, and its fitting over the mathematical model (Figure 4). Data for sfIMT3 showed a smooth transfer function that fit the mathematical model when activated with T3 (Figure 4A) and T4 (Figure 4B). The fitting showed a high correlation between the experimental and modeling results (*r* ^2^ = 0.99). Using a transfer function, the proposed mathematical model can describe the relationship between the measured GFP fluorescence and the corresponding ligand concentration (see Supplementary Section S3.1, 3.1 for details on the derivation of the sfIMT3 model). In contrast, data for dIMT3 showed a smooth transfer function that did not fit the mathematical model, with an inverted Hill function only when the biosensor was induced with T3 (Figure 4C). The amount of T4 was unrelated to the fluorescence measured by dIMT3 (Figure 4D).

### 3.3 sfIMT3 shows similar metrics for T3 and T4

To better understand the performance of the biosensor, several parameters were extracted from their transfer functions (Table 2). When the activity of sfIMT3 was compared for the agonists T3 and T4, the use of T4 as an agonist decreased the amplitude of the biosensor transfer function by approximately 40% (*p* = 3.0 ⋅ 10^–5^, *g* = 1.83). Rather, the SNR values of sfIMT3 indicated that the noise measurement for T3 was negligible compared with that for T4 (*p* = 3.7 ⋅ 10^–1^, *g* = −0.45). Changing the agonist to T4 produced a frameshift in the dynamic range, increasing the maximum and minimum detected concentrations by about one order of magnitude (*p* = 3.8 ⋅ 10^–5^, *g* = −4.44). Thus, the use of T4 as an agonist did not alter the sensitivity of TH measurements using sfIMT3.

**TABLE 2:** Biosensor parameters when induced with T3 or T4.

	OD IMT3 Biosensor Gierach et al. (2012)	sfIMT3 T3 analysis	sfIMT4 T4 analysis	dIMT3 T3 analysis
Amplitude (Fluorescence/OD)	-	1	0.6	22.64
SNR	-	104.77	140.87	363.34
Sensitivity	1.17	0.5	0.5	−0.24
Dynamic range: lower bound (M)	2.11 ⋅ 10^–7^	1.00 ⋅ 10^–7^	9.03 ⋅ 10^–7^	3.38 ⋅ 10^–9^
Dynamic range: upper bound (M)	1.16 ⋅ 10^–6^	5.47 ⋅ 10^–6^	4.93 ⋅ 10^–5^	1.18 ⋅ 10^–5^

### 3.4 dIMT3 shows better metrics than sfIMT3

Relative to sfIMT3, dIMT3 improved the amplitude of the signal (*p* = 4.8 ⋅ 10^–15^, *g* = −16.03; Figure 4C; Table 2). This was followed by a non-significant increase in the SNR of dIMT3 with respect to sfIMT3 (*p* = 3.1 ⋅ 10^–1^, *g* = −0.51). In addition, reducing the Hill coefficient of the dIMT3 sensor relative to that of the sfIMT3 sensor by 50% reduced the sensitivity of dIMT3 by one-half. This reduction in sensitivity allowed for a lower bound of the dynamic range (*p* = 3.8 ⋅ 10^–5^, *g* = −4.44; Table 2).

## 4 Discussion

A quantitative TH biosensor that emits GFP fluorescence proportional to the ligand concentration in culture medium was successfully expressed in *E. coli* cells. The IMT3 biosensor was further optimized using sfGFP as a reporter and adding a double LBD to reduce the lower bound of the dynamic range to physiological levels (7.47 ⋅ 10^–11^–2.87 ⋅ 10^–9^ M for T3 and 1.12 ⋅ 10^–9^–1.61 ⋅ 10^–7^ M for T4) (Dickson, 1990; Cortés-Blanco et al., 1999). sfIMT3 and dIMT3 were also characterized through mathematical models, enabling reliable measurements of TH concentrations.

### 4.1 Engineering cycle for IMT3 biosensor development

The decision to assemble and express the eIMT3, rather than starting directly by characterizing sfIMT3, was justified by studies on splitting eGFP, but not sfGFP (Tornabene et al., 2019). The splicing activity of inteins is known to exhibit a sequence preference at extein residues adjacent to the splice site (i.e., mandatory catalytic Cys, Ser, or Thr residue at the first residue within the C-extein, plus a bias for residues resembling the proximal N- and C-extein sequences found at the native insertion sites) (Amitai et al., 2009; Stevens et al., 2017). Since the optimal splice site of eGFP has already been characterized and the eIMT3 construct was expressed by the cells and reconstituted with functional eGFP after splicing upon binding T3, the sfIMT3 construct was assembled (Tornabene et al., 2019). The similarities between eGFP and sfGFP reconstitution by the IMT3 biosensor suggest that the splicing point can be shared, but the functionality and orthogonality of intein systems should also be fully characterized for the *Mtu* RecA intein construct (Pinto et al., 2020).

The lower boundary of the dynamic range of sfIMT3 was not small enough to detect physiological concentrations of T3 in human blood (Dickson, 1990). An extra THR-*β*_1_ was inserted in tandem with the already present THR-*β*_1_ of sfIMT3, resulting in the assembly of the dIMT3 construct, which reduces the lower bound of the dynamic range. These results are in agreement with a previous finding, showing that increasing the number of receptors amplifies the dynamic range of the biosensor (Gonzalez-Flo et al., 2020). In contrast, the present study did not confirm the results showing that increasing the number of receptors did not affect the sensitivity or length of the dynamic range (Gonzalez-Flo et al., 2020). Comparing the responses of sfIMT3 and dIMT3 showed that receptor duplication increased the length of the dynamic range only by reducing the lower boundary, thus affecting the sensitivity of dIMT3.

Inclusion of a second LBD altered biosensor behavior by inverting its function with respect to sfIMT3, so that it behaved like a different logical circuit. These experimental data could no longer be fitted to the model described in this study but could be fitted to an inverted Hill function. This behavior may be due to the method of folding of the dIMT3 protein chimera, as the addition of the second LBD could lead to conformational changes that mask some of the binding regions of the LBDs, possibly inducing constitutive splicing activity in the *Mtu* RecA intein. Modeling showed that the Hill coefficient was equal to 0.5, indicating negative cooperativity as both binding sites were equal and the ligands could compete and attach to both. Negative cooperativity indicates that the receptor domain loses affinity for T3 when one LBD is occupied by the ligand, possibly indicating that binding of a single LBD to T3 prevents the cleavage of sfGFP and alters the fluorescence intensity.

### 4.2 T3 is a better ligand than T4 for IMT3 biosensors

Owing to the nature of the pituitary-thyroid axis, THR-*β*_1_ binds to both T3 and T4, although its affinity to T3 is higher (Wejaphikul et al., 2019). Because both molecules are present at different concentration ranges in the bloodstream, it is extremely important to characterize the behavior of IMT3 biosensors upon induction with T3 and T4. The concentration of T3 in adult blood ranges from 7.47 ⋅ 10^–11^ to 2.87 ⋅ 10^–9^ M, whereas the concentration of T4 ranges from 1.12 ⋅ 10^–9^ to 1.61 ⋅ 10^–7^ M (Dickson, 1990; Cortés-Blanco et al., 1999). Thus, high affinity of the receptor to T4 would not allow the sensing of T3. Although differences in the binding of THR-*β*_1_ to T3 and T4 might appear merely as a frameshift of the dynamic range to higher concentrations, the results for sfIMT3 suggest that the relationships of THR-*β*_1_ with T3 and T4 differ (Wejaphikul et al., 2019). Rather, the change in agonist from T3 to T4 produced the previously described frameshift, with increases in the maximum and minimum detected concentrations of about one order of magnitude, as well as a sharp decrease in the amplitude of the Hill curve. The SNR of sfIMT3 indicated a non-significant variation of noise in the T3 measurement with respect to T4. This variation in noise may be improved by adding more replicates because the effect size was large (|*g*| > 1). dIMT3 did not show differential splicing activity for T4, indicating that it had greater specificity for T3 than did sfIMT3. This represents an improvement in the ability to identify a specific sensor for T3 without the response of similar ligands, such as T4 (Baluta et al., 2023). Overall, IMT3 biosensors showed better performance with T3 than T4, indicating that T3 is a better agonist for future point-of-care treatments for thyroid diseases.

### 4.3 GFP-based IMT3 biosensors perform better than OD-based biosensors

The sfIMT3 and dIMT3 biosensors showed better performances in sensing THs concentrations than did OD-based IMT3 biosensors (Gierach et al., 2012). The sensitivity of the sfIMT3 biosensors ranged between 0 and 1, indicating that these biosensors were neither too robust, meaning that they would be unable to react to different T3 concentrations, nor hypersensitive, meaning that they would overreact to minimal changes in TH concentrations. In contrast, OD-based IMT3 biosensors show clear hypersensitivity (i.e., *ξ* > 1), reducing their dynamic range.

The present study found that the sensitivity of dIMT3 was lower than that of sfIMT3, thus allowing dIMT3 to react to smaller protein variations in a wider dynamic range with a decreased lower sensing bond. Nevertheless, because the sensitivity of dIMT3 ranged between −1 and 1, the possible negative effects of lower sensitivity on the sensing of THs can be considered negligible. The 50% reduction of the Hill coefficient shown by dIMT3 may have been due to the effect of ligand binding by only one of the two LBDs, a binding that may have been sufficient to affect the dIMT3 splicing activity. Consequently, dIMT3 is a better option for measuring lower protein concentrations, at which differences are less noticeable. Thus, dIMT3 can detect lower protein concentrations around the range observed in the human bloodstream, surpassing the limited sensitivity ranges of current state-of-the-art IMT3 biosensors (Dickson, 1990; Cortés-Blanco et al., 1999; Gierach et al., 2012).

Evaluation of SNR showed that noise was lower with the dIMT3 than with the sfIMT3 biosensor, although the difference was not statistically significant. However, the dIMT3 biosensor showed a greater degree of leakiness. Reductions in leakiness may enable these biosensors to obtain more accurate and precise measurements in actual clinical scenarios.

### 4.4 Conclusion

Thyroid diseases are severe illnesses with a high prevalence worldwide. Reliable tools are needed to avoid the trial-and-error adjustments of treatment doses, as these methods do not ensure proper patient-specific care and drug effectiveness without adverse effects (American Thyroid Association et al., 2004; Chiovato et al., 2019). The present study describes the use of synthetic and systems biology to improve IMT3 biosensors that could help in the diagnosis and treatment of thyroid diseases. Specifically, the sfIMT3 biosensor, which is dependent on protein splicing, not on cell proliferation, is a further step for a future *ex vivo* use (Tinafar et al., 2019). This sfIMT3 was further enhanced by adding a second LBD to its receptor domain, with the dynamic range of the resulting dIMT3 biosensor allowing the measurement of physiological concentrations of T3 at the steady-state (e.g., 30 h in the present *in vitro* experiments), which are features not reported in previous versions of the biosensor. The rationale underlying the lower dynamic range of the dIMT3 biosensor has not been determined, with future studies required to determine its post-translational configuration. Moreover, for clinical applications, the high specificity for physiologic T3 ranges of the dIMT3 biosensor could allow its sensing at the interstitial fluid several times per week through a subcutaneous device, if T3 follows plasma/interstitial fluid concentration ratios similar to other hormones such as cortisol (Dervisevic et al., 2020).

The behavior of the sfIMT3 and dIMT3 biosensors fit almost perfectly with simple mathematical models. Providing biosensors with mathematical models that characterize them facilitates their use in technological applications such as medical devices that can make use of biosensors. Moreover, because the sensing activity is linked to a receptor domain that can be easily exchanged (e.g., LBDs from other receptors), the presented workflow opens the scope for a wide range of biosensor designs that can be readily characterized by the proposed model.

This study establishes the foundation for a novel T3 hormone intein-mediated biosensor with promising clinical applications following the current trends in transdermal diagnostics and drug delivery (Idrees et al., 2019; Dervisevic et al., 2020; Stewart et al., 2021; Mohammadi et al., 2023). The findings of this study indicate that these types of translational projects can result in rapid and proper clinical diagnoses of hormonal disorders.

## Data Availability

The data presented in the study has been deposited in the Figshare repository, accession number 10.6084/m9.figshare.25368127.

## Footnotes

1 iGEM Foundation, Registry of Standard Biological Parts. Registry of Standard Biological Parts (2009). https://parts.igem.org/Main_Page

2 GitHub-Data analysis and plots code (iGEM Barcelona). https://github.com/igemBarcelona/Data-analysis-and-plots-code
