# Supplementary material for: Intein-mediated thyroid hormone biosensors: towards controlled delivery of hormone therapy
Source: Front Syst Biol. 2024 Apr 3;4:1270071. doi: 10.3389/fsysb.2024.1270071 (PMC12342043; doi:10.3389/fsysb.2024.1270071)
Supplement: Supplementary file 1 [file DataSheet1.PDF]

Supplementary Material

1 PLASMIDS AND DNA SEQUENCES

1.1 Plasmids

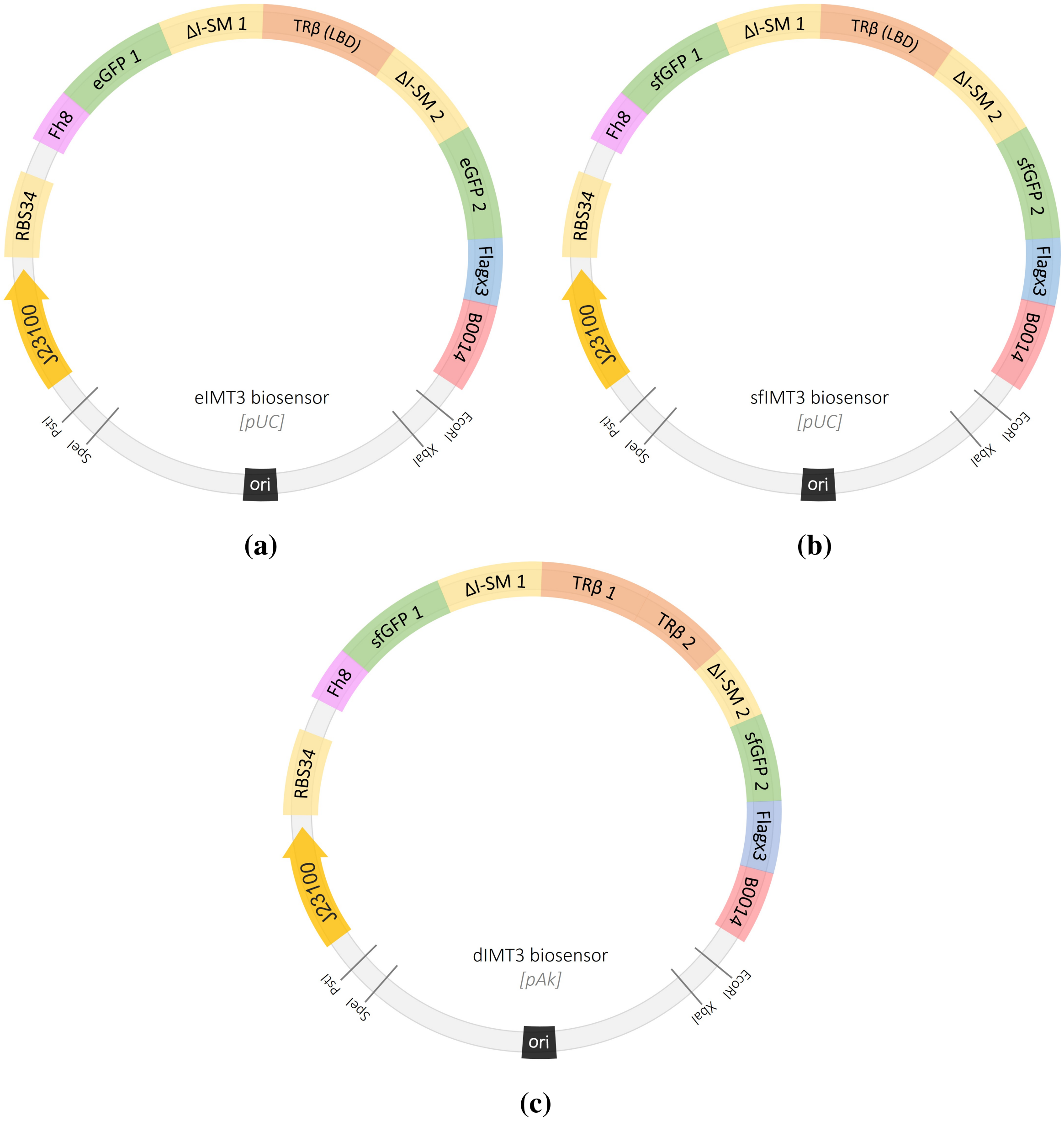

Figure S1: Final constructs of the IMT3 biosensor with **(a)** eGFP, **(b)** sfGFP, and **(c)** double LBD.

1.2 Biosensor DNA Sequences

eIMT3: GAATTCGCGGCCGCTTCTAGAGTTGACGGCTAGCTCAGTCCTAGGTACAGTGCTAG
CTACTAGAGAAAGAGGAGAAAAATAATGCCGTCCGTTCAGGAAGTGGAAAAGCTTTTACA
CGTTCTTGATCGCAACGGCGATGGGAAGGTATCCGCAGAGGAGTTGAAAGCATTCGCAGAC
GATAGCAAATGCCCCTTGGACAGCAACAAAATCAAGGCGTTCATCAAGGAACACGATAAG
AACAAAGACGGAAAATTAGATCTTAAAGAATTAGTATCTATCTTGAGCAGCGTGTCAAAGG
GGGAGGAACTTTTTACCGGAGTTGTGCCCATTCTTGTGGAATTGGACGGTGACGTCAATGG
CCACAAGTTTTCGGTGTCTGGTGAAGGCGAGGGAGATGCCACCTACGGAAATTAACATTG
AAATTTATCTGTACGACAGGGAAGTTGCCCGTGCCCTGGCCAACGCTGGTTACTACGCTTA
CTTACGGTGTTCAGTGTTTGGCCGAGGGTACGCGGATTTTCGATCCCGTGACGGGTACCAC
ACACCGTATCGAAGACGTTGTAGATGGGCGGAAGCCTATCCATGTGGTAGCCGCTGCCAAG
GATGGAACGTTGCACGCGCGCCCTGTAGTATCATGGTTCGATCAAGGGACTCGGGACGTGA
TAGGTCTGCGCATCGCAGGGGGCGCTATACTGTGGGCTACTCCCGACCATAAGGTCTTAAC
GGAGTATGGGTGGCGCGCTGCCGGCGAGTTACGGAAAGGGGACCGCGTGGCCCAACCCCG
GAGATTCGATGGGTTCGGCGACTCAGCACCAATTCCGGCCGAATTACAAAAATCTATTGGT
CACAAGCCGGAGCCTACAGACGAAGAGTGGGAGCTGATAAAGACGGTAACCGAGGCGCAC
GTTGCTACAAACGCACAGGGTTCACATTGGAAACAGAAACGGAAATTCTTACCTGAAGATA
TCGGACAAGCGCCAATAGTGAACGCGCCTGAGGGCGGAAAAGTTGACCTTGAAGCCTTTTC
ACATTTCACAAAAATAATAACACCTGCTATCACGCGCGTGGTGGATTTTGCTAAAAAACTG
CCGATGTTCTGCGAGTTACCGTGTGAGGATCAGATAATCCTTCTGAAGGGCTGTTGTATGG
AAATCATGTCGTTGAGAGCAGCTGTACGCTACGACCCTGAGTCTGAGACTTTGACTCTGAA
CGGGGAGATGGCCGTAACCCGGGGCCAGTTGAAGAATGGGGGTCTTGGTGTAGTCTCCGAT
GCAATCTTTGATCTGGGAATGTCATTAAGTAGTTTTAACTTGGATGACACCGAGGTTGCTTT
ACTTCAGGCGGTTCTTTTAATGAGTAGTGATCGTCCGGGTTTAGCCTGTGTTGAACGCATAG
AAAAGTACCAGGATTCATTTCTGTTGGCTTTTGAGCACTACATCAATTACCGCAAGCATCAC
GTAACGCATTTTTGGCCCAAGCTTTTAATGAAGGTCACCGATCTTCGGATGATAGGCGCCTG
CCACGCGTCACGGTTTTTACACATGAAGGTCGAGTGTCCGACAGAACTTTTCCCCCCACTTT
TTTTAGAGGTGTTCGAGGACCGTGTCCAAGCCTTAGCTGACGCCTTAGATGACAAATTTTTG
CATGACATGCTGGCGGAAGAGCTTAGATATAGCGTCATACGGGAAGTATTGCCCACTAGAC
GGGCGCGTACATTTGATTTAGAGGTAGAAGAACTTCATACTTTGGTCGCCGAAGGCGTCGT
CGTGCACAATTGTTTCAGCCGCTATCCAGATCACATGAAGCAGCACGACTTTTTCAAATCCG
CTATGCCTGAAGGCTATGTTCAAGAGCGCACAATCTTTTTTAAAGATGACGGTAACTATAA
AACGCGCGCAGAAGTTAAGTTTGAAGGAGATACTCTGGTAAACCGGATTGAGTTGAAAGGT
ATCGATTTTAAGGAAGATGGAAATATATTGGGTCATAAGTTGGAGTACAATTATAATTCCC
ACAACGTATATATAATGGCCGATAAACAAAAGAATGGTATTAAAGTGAATTTTAAGATCAG
ACATAATATAGAAGATGGCTCGGTCCAGTTGGCTGACCACTACCAACAGAATACCCCGATT
GGTGACGGACCCGTTCTGTTGCCTGACAATCATTATCTTTCGACACAGAGCGCGTTAAGCA
AAGACCCTAATGAAAAACGGGACCACATGGTTCTGTTGGAGTTCGTGACTGCTGCCGGCAT
CACTCTGGGGATGGACGAACTTTATAAGGATTATAAGGACCACGATGGCGATTACAAGGAT
CATGACATCGATTACAAAGATGACGACGACAAATAATAGTCACACTGGCTCACCTTCGGGT
GGGCCTTTCTGCGTTTATATACTAGAGAGAGAATATAAAAAGCCAGATTATTAATCCGGCT
TTTTTATTATTTTACTAGTAGCGGCCGCTGCAG

sfIMT3: TCATGGTCTCATGCCGAATTCGCGGCCGCTTCTAGAGTTGACGGCTAGCTCAGTCC
TAGGTACAGTGCTAGCTACTAGAGAAAGAGGAGAAAAATAATGCCGTCCGTTCAGGAAGT

GGAAAAGCTTTTACACGTTCTTGATCGCAACGGCGATGGGAAGGTATCCGCAGAGGAGTTG
AAAGCATTCGCAGACGATAGCAAATGCCCCTTGGACAGCAACAAAATCAAGGCGTTCATCA
AGGAACACGATAAGAACAAAGACGGAAAATTAGATCTTAAAGAATTAGTATCTATCTTGA
GCAGCCGTAAAGGCGAAGAGCTGTTCACTGGTGTCGTCCCTATTCTGGTGGAACTGGATGG
TGATGTCAACGGTCATAAGTTTTCCGTGCGTGGCGAGGGTGAAGGTGACGCAACTAATGGT
AAACTGACGCTGAAGTTCATCTGTACTACTGGTAAACTGCCGGTACCTTGGCCGACTCTGGT
AACGACGCTGACTTATGGTGTTCAGTGTTTGGCCGAGGGTACGCGGATTTTCGATCCCGTGA
CGGGTACCACACACCGTATCGAAGACGTTGTAGATGGGCGGAAGCCTATCCATGTGGTAGC
CGCTGCCAAGGATGGAACGTTGCACGCGCGCCCTGTAGTATCATGGTTCGATCAAGGGACT
CGGGACGTGATAGGTCTGCGCATCGCAGGGGGCGCTATACTGTGGGCTACTCCCGACCATA
AGGTCTTAACGGAGTATGGGTGGCGCGCTGCCGGCGAGTTACGGAAAGGGGACCGCGTGG
CCCAACCCCGGAGATTCGATGGGTTCGGCGACTCAGCACCAATTCCGGCCGAATTACAAAA
ATCTATTGGTCACAAGCCGGAGCCTACAGACGAAGAGTGGGAGCTGATAAAGACGGTAAC
CGAGGCGCACGTTGCTACAAACGCACAGGGTTCACATTGGAAACAGAAACGGAAATTCTT
ACCTGAAGATATCGGACAAGCGCCAATAGTGAACGCGCCTGAGGGCGGAAAAGTTGACCT
TGAAGCCTTTTCACATTTCACAAAAATAATAACACCTGCTATCACGCGCGTGGTGGATTTTG
CTAAAAAACTGCCGATGTTCTGCGAGTTACCGTGTGAGGATCAGATAATCCTTCTGAAGGG
CTGTTGTATGGAAATCATGTCGTTGAGAGCAGCTGTACGCTACGACCCTGAGTCTGAGACT
TTGACTCTGAACGGGGAGATGGCCGTAACCCGGGGCCAGTTGAAGAATGGGGGTCTTGGTG
TAGTCTCCGATGCAATCTTTGATCTGGGAATGTCATTAAGTAGTTTTAACTTGGATGACACC
GAGGTTGCTTTACTTCAGGCGGTTCTTTTAATGAGTAGTGATCGTCCGGGTTTAGCCTGTGT
TGAACGCATAGAAAAGTACCAGGATTCATTTCTGTTGGCTTTTGAGCACTACATCAATTACC
GCAAGCATCACGTAACGCATTTTTGGCCCAAGCTTTTAATGAAGGTCACCGATCTTCGGAT
GATAGGCGCCTGCCACGCGTCACGGTTTTTACACATGAAGGTCGAGTGTCCGACAGAACTT
TTCCCCCCACTTTTTTTAGAGGTGTTCGAGGACCGTGTCCAAGCCTTAGCTGACGCCTTAGA
TGACAAATTTTTGCATGACATGCTGGCGGAAGAGCTTAGATATAGCGTCATACGGGAAGTA
TTGCCCACTAGACGGGCGCGTACATTTGATTTAGAGGTAGAAGAACTTCATACTTTGGTCG
CCGAAGGCGTCGTCGTGCACAATTGCTTTGCTCGTTATCCGGACCATATGAAGCAGCATGA
CTTCTTCAAGTCCGCCATGCCGGAAGGCTATGTGCAGGAACGCACGATTTCCTTTAAGGAT
GACGGCACGTACAAAACGCGTGCGGAAGTGAAATTTGAAGGCGATACCCTGGTAAACCGC
ATTGAGCTGAAAGGCATTGACTTTAAAGAAGACGGCAATATCCTGGGCCATAAGCTGGAAT
ACAATTTTAACAGCCACAATGTTTACATCACCGCCGATAAACAAAAAAATGGCATTAAAGC
GAATTTTAAAATTCGCCACAACGTGGAGGATGGCAGCGTGCAGCTGGCTGATCACTACCAG
CAAAACACTCCAATCGGTGATGGTCCTGTTCTGCTGCCAGACAATCACTATCTGAGCACGC
AAAGCGTTCTGTCTAAAGATCCGAACGAGAAACGCGATCATATGGTTCTGCTGGAGTTCGT
AACCGCAGCGGGCATCACGCATGGTATGGATGAACTGTACAAAGATTATAAGGACCACGA
TGGCGATTACAAGGATCATGACATCGATTACAAAGATGACGACGACAAATAATAGTCACA
CTGGCTCACCTTCGGGTGGGCCTTTCTGCGTTTATATACTAGAGAGAGAATATAAAAAGCC
AGATTATTAATCCGGCTTTTTTATTATTTTACTAGTAGCGGCCGCTGCAGGCAATGAGACCA
TGA

dIMT3: TTGACGGCTAGCTCAGTCCTAGGTACAGTGCTAGCTACTAGAGAAAGAGGAGAAA
AATAATGCCGTCCGTTCAGGAAGTGGAAAAGCTTTTACACGTTCTTGATCGCAACGGCGAT
GGGAAGGTATCCGCAGAGGAGTTGAAAGCATTCGCAGACGATAGCAAATGCCCCTTGGAC
AGCAACAAAATCAAGGCGTTCATCAAGGAACACGATAAGAACAAAGACGGAAAATTAGAT
CTTAAAGAATTAGTATCTATCTTGAGCAGCCGTAAAGGCGAAGAGCTGTTCACTGGTGTCG

TCCCTATTCTGGTGGAACTGGATGGTGATGTCAACGGTCATAAGTTTTCCGTGCGTGGCGAG
GGTGAAGGTGACGCAACTAATGGTAAACTGACGCTGAAGTTCATCTGTACTACTGGTAAAC
TGCCGGTACCTTGGCCGACTCTGGTAACGACGCTGACTTATGGTGTTCAGTGTTTGGCCGAG
GGTACGCGGATTTTCGATCCCGTGACGGGTACCACACACCGTATCGAAGACGTTGTAGATG
GGCGGAAGCCTATCCATGTGGTAGCCGCTGCCAAGGATGGAACGTTGCACGCGCGCCCTGT
AGTATCATGGTTCGATCAAGGGACTCGGGACGTGATAGGTCTGCGCATCGCAGGGGGCGCT
ATACTGTGGGCTACTCCCGACCATAAGGTCTTAACGGAGTATGGGTGGCGCGCTGCCGGCG
AGTTACGGAAAGGGGACCGCGTGGCCCAACCCCGGAGATTCGATGGGTTCGGCGACTCAG
CACCAATTCCGGCCGAATTACAAAAATCTATTGGTCACAAGCCGGAGCCTACAGACGAAGA
GTGGGAGCTGATAAAGACGGTAACCGAGGCGCACGTTGCTACAAACGCACAGGGTTCACA
TTGGAAACAGAAACGGAAATTCTTACCTGAAGATATCGGACAAGCGCCAATAGTGAACGC
GCCTGAGGGCGGAAAAGTTGACCTTGAAGCCTTTTCACATTTCACAAAAATAATAACACCT
GCTATCACGCGCGTGGTGGATTTTGCTAAAAAACTGCCGATGTTCTGCGAGTTACCGTGTG
AGGATCAGATAATCCTTCTGAAGGGCTGTTGTATGGAAATCATGTCGTTGAGAGCAGCTGT
ACGCTACGACCCTGAGTCTGAGACTTTGACTCTGAACGGGGAGATGGCCGTAACCCGGGGC
CAGTTGAAGAATGGGGGTCTTGGTGTAGTCTCCGATGCAATCTTTGATCTGGGAATGTCATT
AAGTAGTTTTAACTTGGATGACACCGAGGTTGCTTTACTTCAGGCGGTTCTTTTAATGAGTA
GTGATCGTCCGGGTTTAGCCTGTGTTGAACGCATAGAAAAGTACCAGGATTCATTTCTGTTG
GCTTTTGAGCACTACATCAATTACCGCAAGCATCACGTAACGCATTTTTGGCCCAAGCTTTT
AATGAAGGTCACCGATCTTCGGATGATAGGCGCCTGCCACGCGTCACGGTTTTTACACATG
AAGGTCGAGTGTCCGACAGAACTTTTCCCCCCACTTTTTTTAGAGGTGTTCGAGGACCGTAT
TAGCCAGAGGATGGAGCTGCAGAAGTCGATCGGCCATAAACCTGAACCAACCGATGAGGA
ATGGGAACTTATTAAAACCGTTACGGAAGCACATGTGGCAACGAATGCGCAAGGATCTCAC
TGGAAGCAAAAGCGCAAGTTTCTCCCAGAGGACATTGGGCAGGCACCGATTGTTAATGCCC
CGGAAGGTGGCAAGGTGGATCTCGAGGCGTTCAGCCACTTTACCAAGATTATCACCCCGGC
AATTACCCGTGTAGTCGACTTCGCAAAGAAGCTTCCCATGTTTTGTGAACTGCCTTGCGAAG
ACCAAATCATTCTGCTTAAAGGGTGCTGCATGGAGATTATGAGTCTCCGTGCGGCCGTGCG
TTATGATCCGGAATCAGAAACACTGACCCTCAATGGTGAAATGGCTGTGACGCGTGGTCAA
CTGAAGAACGGCGGCCTCGGCGTGGTGTCGGACGCGATTTTCGACCTCGGGATGAGTTTGA
GCTCCTTCAATTTAGACGATACGGAAGTGGCCCTGCTGCAAGCCGTGCTGCTCATGAGCTCT
GACCGCCCAGGACTGGCGTGCGTGGAGCGTATTGAGAAATATCAAGACTCGTTCTTATTAG
CATTCGAACATTATATTAACTATCGTAAACACCATGTCACCCACTTCTGGCCGAAACTGCTG
ATGAAAGTGACTGACCTGCGCATGATCGGAGCTTGTCATGCTAGCCGCTTCCTGCATATGA
AAGTGGAATGCCCCACGGAGCTGTTTCCGCCGTTATTCCTCGAAGTATTTGAAGATGCTAG
CTGTGGGATGCGTGTCCAAGCCTTAGCTGACGCCTTAGATGACAAATTTTTGCATGACATGC
TGGCGGAAGAGCTTAGATATAGCGTCATACGGGAAGTATTGCCCACTAGACGGGCGCGTAC
ATTTGATTTAGAGGTAGAAGAACTTCATACTTTGGTCGCCGAAGGCGTCGTCGTGCACAATT
GCTTTGCTCGTTATCCGGACCATATGAAGCAGCATGACTTCTTCAAGTCCGCCATGCCGGAA
GGCTATGTGCAGGAACGCACGATTTCCTTTAAGGATGACGGCACGTACAAAACGCGTGCGG
AAGTGAAATTTGAAGGCGATACCCTGGTAAACCGCATTGAGCTGAAAGGCATTGACTTTAA
AGAAGACGGCAATATCCTGGGCCATAAGCTGGAATACAATTTTAACAGCCACAATGTTTAC
ATCACCGCCGATAAACAAAAAAATGGCATTAAAGCGAATTTTAAAATTCGCCACAACGTGG
AGGATGGCAGCGTGCAGCTGGCTGATCACTACCAGCAAAACACTCCAATCGGTGATGGTCC
TGTTCTGCTGCCAGACAATCACTATCTGAGCACGCAAAGCGTTCTGTCTAAAGATCCGAAC
GAGAAACGCGATCATATGGTTCTGCTGGAGTTCGTAACCGCAGCGGGCATCACGCATGGTA

TGGATGAACTGTACAAAGATTATAAGGACCACGATGGCGATTACAAGGATCATGACATCGA TTACAAAGATGACGACGACAAATAATAGTCACACTGGCTCACCTTCGGGTGGGCCTTTCTG CGTTTATATACTAGAGAGAGAATATAAAAAGCCAGATTATTAATCCGGCTTTTTATTATTT TACTAGTAGCGGCCGCTG

1.3 Golden Gate Primers

GG-1-fwd plasmid: CGCATGGAATTCTACGGTCTCAACTAGCTGCGTTGACGGCTAGCTCAGT CCT

GG-1-rev plasmid: GCGTATCTGCAGGGTCTCGTCTGGCTAATACGGTCCTCGAACACCTCTA

GG-2-fwd plasmid: CGCATGGAATTCTAGGTCTCACAGAGGATGGAGCTGCAGAAGTCGATC GG

GG-2-rev plasmid GCGGCTCTGCAGGGTCTCGCACAGCTAGCATCTTCAAATACTTCGAGGA ATAACGGC

GG-3-fwd plasmid CGCATGGAATTCTAGGTCTCATGTGGGATGCGTGTCCAAGCCTTAGCTGA

GG-3-rev plasmid: GCGTATCTGCAGCATCGGGTCTCGGTAAGCTAATCAGCGGCCGCTACTA GTAAA

2 TIME DYNAMICS OF IMT3 BIOSENSORS

In this section, the dynamics of T3 fluorescence over time are described for the different intein-mediated biosensor inductors.

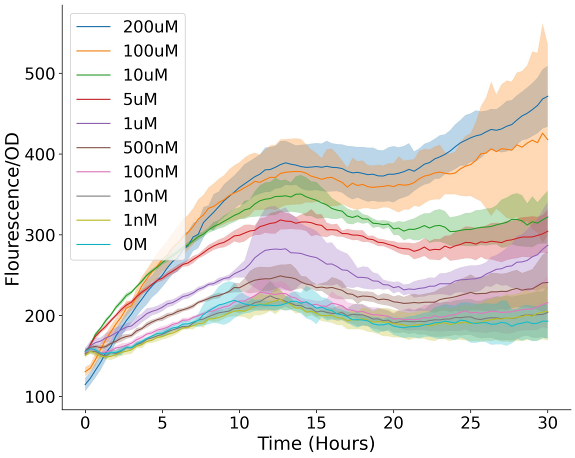

(a)

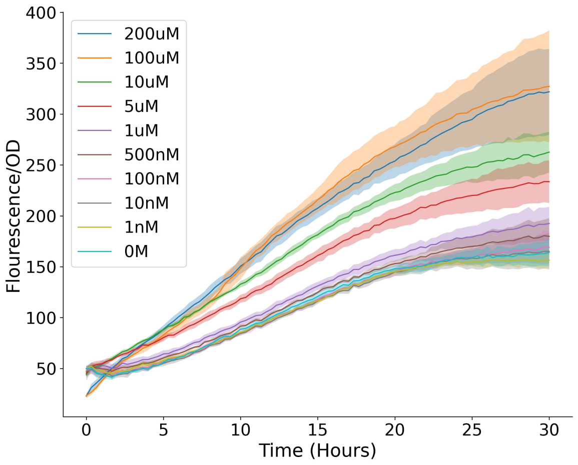

(b)

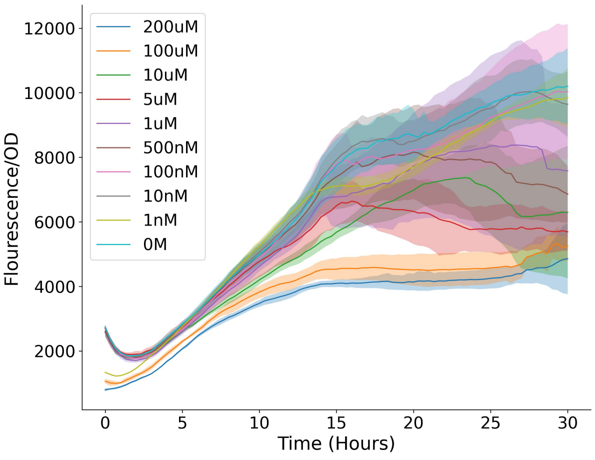

(c)

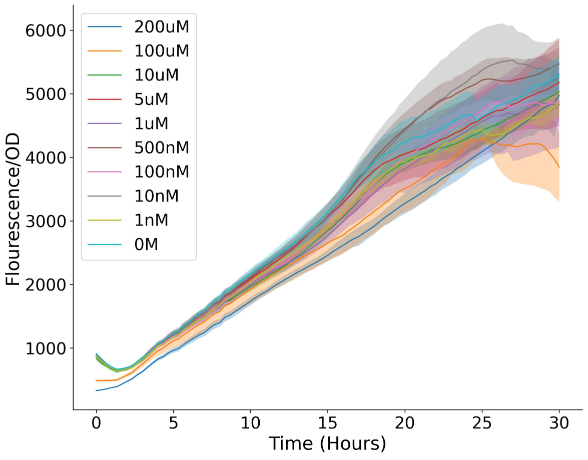

(d)

Figure S2: Dynamic response of sfIMT3 and dIMT3 at different **(a, c)** T3 and **(b, d)** T4 concentrations, respectively. Experimental data consists of the mean and standard deviation of 6 replicates per TH concentration and time step. Cells were grown in fresh LB media + Carbenicillin 25 μg/ml overnight until they attained an OD_{600} of 0.4-0.6. A 100 μL aliquot of cultured cells was added to each well of a 96 well black/clear bottom plate, along with 100 μL of the 2x ligand solutions.

3 MODELLING OF THE INTEIN-MEDIATED BIOSENSOR

Hormonal biosensors are a crucial aspect of the project. The presented intein-mediated biosensor works at the post-translational level and is mediated by inteins, which are proteins that carry out protein splicing. When the hormone binds to the receptor, it splices and reconstructs the split fluorescent protein at two end sites. Therefore, the fluorescent protein is only active when splicing of the intein occurs, that is, if the hormone is present in the medium. As this biological agent can translate biological information into numerical values, we can sense the amount of thyroid hormones (TH) in the medium. Consequently, a model was developed to understand the biosensor behavior and characterize it.

3.1 System Reactions

We can develop six reactions from the system interactions, which form the basis for our intein-based sensor cell model. In general, our biosensors work as follows: a cell produces a receptor protein P at the rate α_P. P is degraded at a rate δ_P, but its free form also vanishes upon binding with ligand A (TH) with β_1 rate, conforming to the precursor form $[PA]$ of the reporter protein R (green fluorescent protein, GFP). PA achieves its functional state R at rate β_2. R is also subject to basal production and degradation at rates α_R and δ_R, respectively. Thus, the parameter β ($\beta = \beta_1\beta_2$) is the production rate of R.

$$
\begin{gathered}
\text{Ø} \xrightarrow{\alpha_P} \text{P} \\
\text{P} \xrightarrow{\delta_P} \text{Ø} \\
\text{P + A} \xrightarrow{\beta_1} \text{[PA]} \\
\text{[PA]} \xrightarrow{\beta_2} \text{R} \\
\text{Ø} \xrightarrow{\alpha_R} \text{R} \\
\text{R} \xrightarrow{\delta_R} \text{Ø}
\end{gathered}
$$

3.2 Ordinary Differential Equations (ODEs)

From the reactions above, we developed a model considering the basality in the production of fluorescent proteins. Moreover, we consider that the variable P always refers to the entire intein construct, including the split fluorescent protein at the end sites. Therefore, when a fluorescent protein was generated, the construct was degraded. This makes sense because the residual construct is non-functional since it has already spliced R. Furthermore, as previously mentioned, it is necessary to multiply the production of R (β_1) at a certain rate (β_2), as the reconstruction can lead to a nonfunctional R. The final ODE system and its parameters for the different inputs can be seen below (Equation S1, S2 $\&$ Table S1). The parameters shown were fitted with normalized fluorescence.

$$
\frac{dP}{dt} = \alpha_P - \beta_1 PA - \delta_P P \tag{S1}
$$

$$
\frac{dR}{dt} = \alpha_R + \beta_2\beta_1 PA - \delta_R R = \alpha_R + \beta PA - \delta_R R \tag{S2}
$$

Table S1. Experimental values of the biosensor model with different inductors of the sfIMT3.

	Units	sfIMT3 T3 analysis	sfIMT3 T4 analysis
α_P	(a.u.)/time	0.943	0.655
α_R	RFU/time	$5.64 \cdot 10^{-5}$	$1.37 \cdot 10^{-5}$
β_1	1/(time·(a.u.))	0.979	0.786
β	1/(time·(a.u.))	0.943	0.656
δ_P	1/time	$7.24 \cdot 10^{-7}$	$5.24 \cdot 10^{-6}$
δ_R	1/time	0.914	0.912
R^2	-	0.99	0.99

3.3 System Transfer Function

From the ODE system, a transfer function can be derived if P and R are in a steady-state ($\frac{dy}{dx} = 0$). Therefore, we first isolated P from the second ODE (Equation S2).

$$P = \frac{\delta_R R - \alpha_R}{\beta A} \tag{S3}$$

Then, we substitute the obtained P (Equation S3) in the other ODE.

$$\frac{dP}{dt} = \alpha_P - \beta_1 \frac{\delta_R R - \alpha_R}{\beta A} A - \delta_P \frac{\delta_R R - \alpha_R}{\beta A} \tag{S4}$$

We isolated R to obtain the transfer function (Equation S5).

$$R^* = \frac{\delta_P \alpha_R + A(\alpha_P \beta + \beta_1 \alpha_R)}{\beta_1 \delta_R A + \delta_P \delta_R} \tag{S5}$$

3.4 Amplitude

To retrieve the maximum and minimum R values, the limits approaching 0 and ∞ must be determined (Equations S6, S7).

$$R_- = \lim_{A \to 0} R = \frac{\alpha_R}{\delta_R} \tag{S6}$$

$$R_+ = \lim_{A \to \infty} R = \frac{\alpha_P \beta + \alpha_R \beta_1}{\delta_R \beta_1} \tag{S7}$$

Then, the amplitude of a biosensor can be described as the difference between the maximum and minimum output values (Equation S8).

$$\gamma = \frac{\alpha_P \beta}{\delta_R \beta_1} \tag{S8}$$

3.5 Biosensor Sensitivity

As we are developing a sensor, it is vital to determine properties that detail its ability to detect and react to changes in the measured physical quantity. To do so, we calculated the sensitivity ξ. Sensitivity is defined as the minimum value of an input parameter that is capable of creating a detectable output change. In our case, it was the minimum change in agonist concentration that generated a difference in the reporter protein concentration, expressed in relative fluorescence units (RFU). To calculate the sensitivity, we used its mathematical definition (Equation S9), which states that the sensitivity is a derivative normalized to the input and output.

$$\xi = \frac{dR^*}{dA}\frac{A}{R} = \frac{A}{R}\frac{\alpha_P \delta_P \beta}{\delta_R(\delta_P + \beta_1 A)^2} \tag{S9}$$

3.6 Dynamic Range

In order to get the limits for the dynamic range, a tangent line that crosses the point where the input value produces half the maximum output value was calculated ($K_{0.5} = \frac{\delta_P}{\beta_1}$) (Gonzalez-Flo et al., 2020). The R value at this point is given by Equation S10.

$$R_{0.5} = \frac{\alpha_P \beta}{2\delta_R \beta_1} + \frac{\alpha_R}{\delta_R} \tag{S10}$$

To obtain the slope of the transfer function (Equation S5), the derivative of the transfer function with respect to $C = log(A)$ at point $K_{0.5}$ must be obtained (Equation S11-S13).

$$\frac{dR}{dC} = \frac{dR}{dA}\frac{dA}{dC} \tag{S11}$$

$$M = \frac{dR}{dC}_{K_{0.5}} \tag{S12}$$

$$M = \frac{\alpha_P \beta ln(10)}{4\delta_R \beta_1} \tag{S13}$$

If the tangent line is defined as $f = M \cdot C + N$, and it crosses point $K_{0.5}$, the value N can be retrieved ($N = R_{0.5} - M \cdot K_{0.5}$) (Equation S14).

$$N = \frac{\alpha_P \beta}{2\delta_R \beta_1} + \frac{\alpha_R}{\delta_R} - \frac{\alpha_P \beta ln(10)}{4\delta_R \beta_1} log\left(\frac{\delta_P}{\beta_1}\right) \tag{S14}$$

Finally, the tangent line is given by (Equation S15).

$$f = \frac{\alpha_P \beta ln(10)}{4\delta_R \beta_1} C + \frac{\alpha_R}{\delta_R} + \frac{\alpha_P \beta}{2\delta_R B}\left(1 - \frac{ln(10) log(\frac{\delta_P}{\beta_1})}{2}\right) \tag{S15}$$

Then, the upper (C_+) and lower (C_-) bounds are defined as the points at which the tangent line intersects the maximum and minimum output values of the biosensor (Equation S16, S17).

$$C_- = log\left(\frac{\delta_P}{\beta_1}\right) - \frac{2}{ln(10)} \tag{S16}$$

$$C_+ = log\left(\frac{\delta_P}{\beta_1}\right) + \frac{2}{ln(10)} \tag{S17}$$

3.7 Stability Analysis

The stability of the system is an important metric to determine if our system will converge to this steady-state (stable) or diverge from it (unstable). To obtain the Jacobian (J), we must perform the derivative of the system ODE ($f(t) = \frac{dP}{dt}$, $g(t) = \frac{dR}{dt}$). From J, we can compute the trace [$tr(J)$] and determinant [$det(J)$] so that we can analyze the stability of the system.

$$J = \begin{bmatrix} \frac{df}{dP} & \frac{df}{dR} \\ \frac{dg}{dP} & \frac{dg}{dR} \end{bmatrix} = \begin{bmatrix} -\beta_1 A - \delta_P & 0 \\ \beta A & -\delta_R \end{bmatrix} \tag{S18}$$

$$det(J) = (-\beta_1 A - \delta_P)(-\delta_R) = \beta_1 A \delta_R + \delta_P \delta_R \tag{S19}$$

$$tr(J) = -\beta_1 A - \delta_P - \delta_R \tag{S20}$$

As $\beta_1, \delta_P, \delta_R > 0$, the determinant (Equation S19) is positive and the trace (Equation S20) is negative, which confirms that the system has a stable steady-state. This means that even if the system is far from the steady-state, for example, when we include an agonist in the media and the biosensor protein has not yet been formed, the system will evolve towards the steady-state, thus sensing the agonist concentration.

3.8 Fitting to Hill Functions

As the previously constructed model can only work in single-receptor, direct-sensing, intein-mediated biosensors with a hill number close to 1, the data from the optical density (OD), the superfolder intein-mediated T3 (sfIMT3) and the double intein-mediated T3 (dIMT3) biosensors must be fitted with two different hill functions (Equations S21, S22) (Gierach et al., 2012).

$$R = \frac{BA^n}{K^n + A^n} + C \tag{S21}$$

$$R = \frac{B}{1 + (A/K)^n} + C \tag{S22}$$

Table S2. Hill function parameters of different intein-mediated biosensors.

	Units	OD intein-mediated T3 biosensor (Gierach et al., 2012)	dIMT3 T3 analysis
B	(a.u.)/time	1	22.637
K	$RFU/time$	$4.96 \cdot 10^{-7}$	$2.00 \cdot 10^{-7}$
n	$1/(time \cdot (a.u.))$	2.34	0.490
C	$1/(time \cdot (a.u.))$	0	0.433

4 DMSO DOSE-RESPONSE CURVES

To assess the potential impact of DMSO on the biosensor's functionality, we conducted a plate-reader analysis. This analysis involved measuring OD_660 and emission at 517 nm in wells containing LB media, Carbomycin, and varying concentrations of DMSO, with or without cells (sfIMT3 or dIMT3 cells). In Table S3, the DMSO concentrations corresponding to the T3 concentrations in the original plate-reader data are presented. Figure S3a shows that DMSO had minimal effect on both media autofluorescence and OD_660 ($r = -0.39$ and $r = -0.05$, respectively). Furthermore, DMSO had only a slight impact on the dose-response curves for T3 versus GFP fluorescence ($r = -0.56$ and $r = -0.69$, respectively). This observation is reinforced by comparing the original T3 dose-response curves for dIMT3 and sfIMT3, which demonstrate their lack of similarity ($p = 0.002$ and $p = 0.004$, respectively).

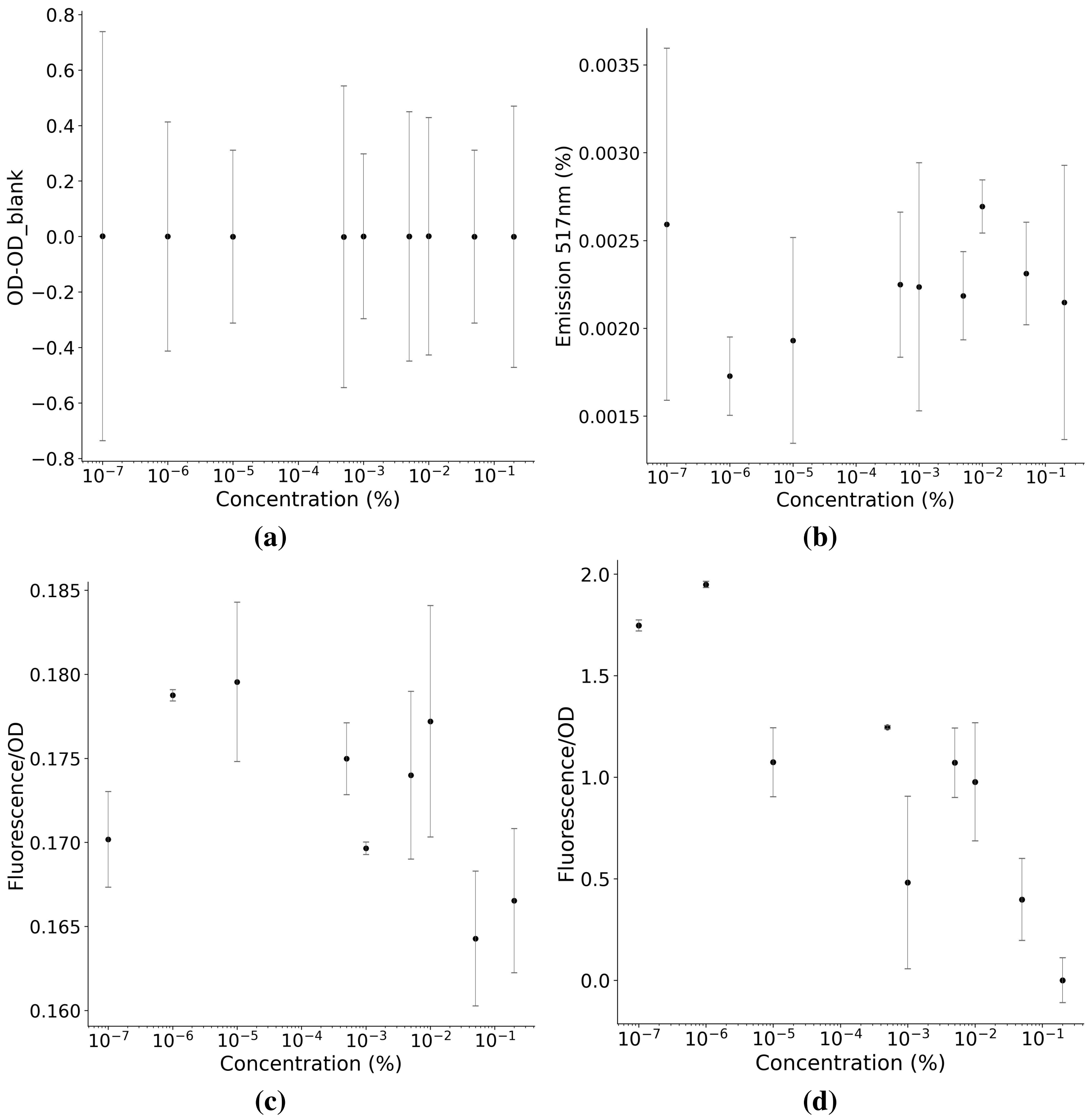

Figure S3: Dose-response curves regarding the effect of DMSO **(a)** on OD (no cells), **(b)** emission 517nm (no cells), **(c)** emission 517nm (sfIMT3), **(d)** emission 517nm (dIMT3).

5 EXPERIMENTAL PROTOCOLS

5.1 Electrocompetent cells

Protocol for the preparation of electro-competent cells for the use of electroporation, a widely employed method for transforming DNA extracts into cells through the application of electric pulses.

Materials:

- BL21 *E. coli* Strain with Antibiotic resistance
- LB media

Table S3. T3/T4 vs DMSO concentrations on plate-reader analysis.

T3 concentration (M)	DMSO concentration (V/V, %)
$2 \cdot 10^{-4}$	$4 \cdot 10^{-1}$
$1 \cdot 10^{-4}$	$2 \cdot 10^{-1}$
$2 \cdot 10^{-5}$	$2 \cdot 10^{-2}$
$5 \cdot 10^{-6}$	$1 \cdot 10^{-2}$
$1 \cdot 10^{-6}$	$2 \cdot 10^{-3}$
$5 \cdot 10^{-7}$	$1 \cdot 10^{-3}$
$1 \cdot 10^{-7}$	$2 \cdot 10^{-4}$
$1 \cdot 10^{-8}$	$2 \cdot 10^{-5}$
$1 \cdot 10^{-9}$	$2 \cdot 10^{-6}$
0	0

- LB + Antibiotic agar plates
- Antibiotic
- Sterile ice-cold distilled water
- Sterile ice-cold distilled 10% glycerol
- 50 mL centrifuge tubes
- Erlenmeyer culture flask
- Ice
- 1.5 mL microcentrifuge tubes
- Shaking incubator
- Bunsen burner
- Spectrophotometer
- Microcentrifuge (for 1.5 mL tubes)
- Centrifuge (for 50 mL tubes)

Procedure:

1. Streak the *E. coli* strain onto an LB + Antibiotic agar plate to isolate single colonies and incubate at 37 ºC overnight.
2. Inoculate 2 mL of LB medium + Antibiotic with a single colony of freshly grown *E. coli* and incubate overnight at 37 ºC with vigorous shaking.
3. Inoculate 50 mL of LB medium with a 1% concentration of resistance (achieved by adding 500 µL of saturated culture) in an Erlenmeyer culture flask. Incubate at 37 ºC with vigorous shaking until the optical density at 600 nm (OD_{600}) is between 0.4 and 0.6.

   Optimal transformation efficiencies are achieved when *E. coli* cells reach an optical density, OD_{600}, between 0.4 and 0.6, resulting in 200 μL of competent cells (using 50 μL for each transformation). To obtain this biomass, the cell suspension should be monitored every 30 minutes using a spectrophotometer. Typically, it takes between

2 and 3 hours after culture inoculation to reach this point.

4. All the following steps are performed under sterile conditions. Transfer the contents from the culture flask to a 50 mL centrifuge tube and place it on ice for 5-10 minutes, maintaining sterility.
5. Pellet the cells by centrifugation at 4600 g for 7 minutes at 4 ºC, and then discard the supernatant.
6. Resuspend the cells in 1 mL of ice-cold sterile distilled water. Then add 30 mL of ice-cold sterile distilled water and centrifuge again.

   It is crucial to keep the cells on ice from this point forward to achieve high transformation efficiency. An increase in cell temperature will result in lower transformation efficiencies.

7. Repeat step 6 until the cells have been washed with water at least three times.

   The cells must be washed extensively in sterile distilled water to remove the growth medium, which may not be suitable for electroporation; for example, it may have a high salt concentration, which will result in sample arcing.

8. Resuspend the cells in 1 mL of ice-cold 10% glycerol and transfer them to a 1.5 mL microcentrifuge tube. Centrifuge at 10,000 rpm for 30 seconds at 4 ºC.
9. Discard the supernatant and repeat step 8.
10. Resuspend the cells in a final volume of 200 μL of ice-cold 10% glycerol.
11. If cells are to be used immediately, place them on ice. To store electrocompetent cells, aliquot them into 50 μL amounts and immediately snap-freeze using a liquid nitrogen bath, then store at -80ºC.

5.2 Electroporation Transformation

Electroporation is a widely used method for transformation of DNA extracts into cells. It is based on sending electrical pulses to the cell, so its polarized membrane forms reversible transient pores that let the DNA flow into the cell. This protocol explains how to transform electro-competent cells via electroporation. This protocol is adapted from Woodall's work (Woodall, 2003).

Materials:

- Electrocompetent *E. coli*
- LB + Antibiotic agar plates
- S.O.C. media
- Ice
- 1.5 mL microcentrifuge tubes
- Electroporation cuvettes
- Electroporation apparatus
- Shaking incubator

Procedure:

1. Remove a 50 μL aliquot of cells from -80 ºC storage and place them directly onto ice to thaw. Once thawed, do not handle the cells vigorously, as this will reduce the transformation efficiency, or use freshly prepared ice-cold electro-competent cells.
2. (Optional) Aliquot 50 μL of cells into an ice-cold electroporation cuvette (0.1 cm gap). Test an aliquot of cells to check that the sample does not arc, using the electroporation conditions in step 6. If arcing does occur, wash cells in 1 mL of ice-cold sterile distilled water and resuspend in 50 μL ice-cold sterile 10% glycerol until arcing does not occur.
3. To the 50μL cell aliquot, add prechilled plasmid DNA (between 5 pg and 100 ng) in a low volume ($< 5\mu$L). Mix by gentle tapping and incubate on ice for 10 to 30 minutes.
4. Place the 0.1 cm electroporation cuvette in ice to chill.
5. Place all the DNA/cell mix into the prechilled electroporation cuvette, making sure it has no bubbles. Then, place the electroporation cuvette into the apparatus.

   IMPORTANT: wipe the sides of the cuvette, making sure it's completely dry.

6. Set the electroporation conditions on a Bio-Rad Gene Pulser to 1.8 KV, 25 μF, and 200 Ω. These conditions may change depending on the bacterial strain and the pulse apparatus; check beforehand. To deliver an electric pulse, press the pulse button until a beep sounds and a time constant appears in the apparatus window. If there is a popping sound, the sample has arced, probably because the plasmid DNA has too high a salt concentration.

   In *E. coli*, time constants should be between 4.5 and 5; lower values probably mean a very low transformation or cell death.

7. Immediately after cells have been pulsed, add 400 μL of room temperature S.O.C. medium and gently resuspend the cells. Transfer the cells to a 1.5 mL microcentrifuge tube and incubate at 37 ºC with vigorous shaking for 1 hour.
8. Spread aliquots of the cells onto LB agar plates containing an antibiotic appropriate for the selection of transformants. Several different dilutions of the cell suspension should be spread onto the plate to obtain single colonies.

If a high number of transformants is expected, the original aliquot of cells can be diluted. One in ten serial dilutions of 100 μL should be spread onto plates. If a low number of transformants is expected, it is better to spread aliquots of 200 μL onto five plates rather than the whole 1 mL on one agar plate to avoid growth inhibition because of dead cells.

5.3 Western Blot

Protocol for Western Blot procedure and the sample preparation. With this technique we can detect the presence of the desired protein. To quantify it, we must apply the SDS-PAGE protocol.

Materials:

- Cell lysis
- NuPAGE® Bis-Tris Gels (Invitrogen)
- Novex Tris-Acetate SDS Running Buffer (20X)
- Blotting Pads
- PVDF Membrane
- XCell II™ Blot Module (Invitrogen)
- XCell SureLock® Mini-Cell gel running tank (Invitrogen)
- TBS (10X) (Axil)
- Tween-20
- Milk (Common stock)
- Primary & secondary antibodies
- Sodium azide (Life)
- Pierce™ ECL Western Blotting Substrate
- Plastic sheet
- BIORAD ChemiDoc™ Imaging System

Procedures:

Boiling Sample Preparation

1. In a 1.5 mL microcentrifuge tube mix 20 μL of cell lysis with 5 μL of loading buffer for each sample.
2. Boil the mix at 95 ºC for 5 minutes in the same microcentrifuge tube.
3. Spin down at maximum speed for 1 minute.
4. Loading buffer should be added to the samples under the hood.

Urea Sample Preparation

1. In a 1.5 mL microcentrifuge tube mix 20 μL of cell lysis with 10 μL of loading buffer and 10 μL of urea for each sample.
2. Incubate the mix at 37 ºC for 15 minutes in the same microcentrifuge tube.
3. Spin down at maximum speed for 1 minute.
4. Loading buffer should be added to the samples under the hood.

Set up Gel Tank:

1. Remove the white tape near the bottom of the polyacrylamide gel and place it in the cuvette.
2. Fill the tank with running buffer and once filled get rid of the comb to expose the wells.
3. Load samples.

4. Connect the tank to the power supply and run the gel at 120 V until the front dye is at the very bottom of the gel and the ladder has separated enough.

Dry Transferring:

1. Once the gel has run, with a spatula, break the plastic case of the gel by carefully inserting the spatula in the lateral grooves and prying.
2. Cut the gel well fringes and the excess gel. Place the gel in a cuvette and wash it with distilled water for a few seconds.
3. Open the iBlot™ 2 dry transfer kit and place the bottom layer with plastic into the iBlot™ 2.
4. Lay down the transfer paper, use rolling pin to avoid any bubble.
5. Lay down the running gel centred, use rolling pin to avoid any bubble.
6. Wet-activate the paper layer in distilled water and lay it on.
7. Put the last metal layer and the filter making sure that the anode and cathode of the layers are touching its respective parts of the machine.
8. Run the iBlot™ 2 with the 7 minutes program.
9. Discard everything but the transfer paper. Mark the top side with a pencil.
10. Cut the sides of the paper (without touching the transferred region) so that it fits into the cuvette.

Ponceau staining:

1. Place the transfer paper in a cuvette and add the Ponceau staining solution (reusable).
2. After a couple of minutes, proteins will begin to stain. If that occurs, cell lysis performed correctly.
3. Return the Ponceau staining solution to the original 50 mL centrifuge tube.

Fixing and Antibody incubation:

1. Prepare 1 L of TBST (in our case 500 mL).
2. In a moving cuvette, wash the transfer paper 3 times during 5 minutes changing the TBST in between.
3. Prepare the fixing solution (TBTS 4% milk) by adding 40 mL of TBST in a 50 mL centrifuge tube, and then adding 1.6 g of milk powder (4%) and mixing well.
4. Incubate, in a moving cuvette, the transfer paper with the fixing solution for 15 minutes.
5. Discard the liquid and wash, in a moving cuvette, the transfer paper 3 times during 5 minutes changing the TBST in between.
6. Add the primary antibody solution (10 mL of the TBST 4% milk with primary antibody 1:5000 proportion).
7. Incubate, in a moving cuvette, for 4 hours (or overnight at 4 ºC) with the primary antibody.
8. Wash, in a moving cuvette, the transfer paper 3 times during 5 minutes changing the TBST in between.
9. Add the secondary antibody solution (10 mL of the TBST 4% milk with primary antibody 1:1000 proportion).
10. Incubate, in a moving cuvette, for 1 hour with the secondary antibody.
11. Wash, in a moving cuvette, the transfer paper 3 times during 5 minutes changing the TBST in between.

Western Developing:

1. Prepare the cassette – line interior with an A4 transparent plastic wallet cut to size, tape to hold.
2. Prepare the ECL reagent – Pipette equal volumes of solutions A & B of the ECL reagent into a clean 1.5 mL microcentrifuge tube. Mix by pipetting. For 1 membrane, a total volume of 500 μL is enough. Do not leave mixed reagent sitting for too long, exposure signal will be lost.
3. Remove the membrane from 1X TBS and dab on paper towel to remove excess buffer.
4. Place the membrane, the right way up, on the bottom layer of plastic sheet lining the cassette.
5. Pipette mixed ECL reagent and dispense over membrane.
6. Lower the top layer of plastic sheet and flatten it over the membrane to remove air bubbles and excess solution.
7. Wipe dry spill over at the edges.
8. Remove all air bubbles from membrane.
9. Close the cassette lid and proceed with exposure in the dark room.
10. Wait for 6 minutes and develop with the imaging system.

REFERENCES

Gierach, I., Li, J., Wu, W.-Y., Grover, G. J., and Wood, D. W. (2012). Bacterial biosensors for screening isoform-selective ligands for human thyroid receptors α-1 and β-1. *FEBS Open Bio* 2, 247–253. doi:10.1016/j.fob.2012.08.002

Gonzalez-Flo, E., Alaball, M. E., and Macia, J. (2020). Two-component biosensors: Unveiling the mechanisms of predictable tunability. *ACS Synthetic Biology* 9, 1328–1335. doi:10.1021/acssynbio.0c00010

Woodall, C. A. (2003). Electroporation of e. coli. *Methods Mol Biol.* 235 (55–69). doi:10.1385/1-59259-409-3:55
